# Towards more reliable non-linear Mendelian randomization investigations

**DOI:** 10.1007/s10654-024-01121-9

**Published:** 2024-05-25

**Authors:** Stephen Burgess

**Affiliations:** 1 MRC Biostatistics Unit, University of Cambridge, Cambridge; 2 Cardiovascular Epidemiology Unit, Department of Public Health and Primary Care, University of Cambridge, Cambridge

Non-linear Mendelian randomization (NLMR) is the use of genetic variants as instrumental variables to make inferences about the shape of the causal relationship between an exposure and an outcome at different levels of the exposure^1,2^. Learning the shape of a causal relationship is a natural enquiry that has clear relevance to clinical and epidemiological practice. However, this is not a standard output from a Mendelian randomization investigation (or equivalently, a randomized controlled trial), which typically estimates an average causal effect^1^. Stratification is an appealing solution to this problem. However, stratification on any post-randomization measurement risks breaking randomization^3^. This includes stratification on levels of the exposure in Mendelian randomization, as the genetic “randomization” is determined at conception and so the exposure is a post-randomization measurement. Formally speaking, this is an example of collider bias^4^: the exposure is a common effect of the genetic instrument and exposure—outcome confounders, and hence stratification on the exposure induces an association between the instrument and confounders.

In 2014, we published a method for NLMR that aims to enable stratification using the exposure without inducing collider bias^5^. We refer to this as the “residual stratification” method. In addition to the standard instrumental variable assumptions in Mendelian randomization, this method assumes that genetic effects on the exposure are constant in the population. This assumption is strict and potentially implausible^6^. Subsequently, we developed a further method for NLMR, that we refer to as the “doubly-ranked” method^7^. This method also makes an additional assumption, but a strictly weaker one to the “constant genetic effect assumption”: that genetic effects on the exposure may vary, but they do not change the ranking of individuals according to their level of the exposure. That is, the ordering of all individuals’ exposure values would be the same if their genotypes were set to any value. We refer to this as the “rank-preserving assumption”. As part of scientific due diligence, we re-ran NLMR analyses that we had previously performed using the residual method. For the effect of vitamin D on cardiovascular disease and mortality, we obtained strikingly different answers using the doubly-ranked method. After a long process of consideration and consultation, we retracted the vitamin D work and republished using the updated doubly-ranked method^8^. A major reason for this was a simulation study showing that estimates from the residual stratification method could be seriously biased when the genetic effects on the exposure vary in the population, as they do in the case of vitamin D^6^.

The work in Hamilton *et al* presents a further serious challenge to the validity of NLMR^9^. The authors show that genetic associations with age and sex can appear in strata of the population, even if they do not appear in the population as a whole. This is contrary to what has been seen in simulation studies for the doubly-ranked method, indicating that this appears to be a problem with certain applications of the method, rather than a fault of the method itself. Two potential explanations for this are violation of the rank-preserving assumption, and selection bias. The investigation of Hamilton *et al* is limited in scope: they show associations with age and sex, but not with other variables, and all analyses are conducted in UK Biobank. Associations with other variables have been tested elsewhere, and are typically either absent or less strong^10^. However, other variables are less clear as negative control outcomes; associations with such variables could be due to pleiotropy or true causal effects of the exposure, whereas it is impossible for age and sex to be affected by environmental exposures or autosomal genetic variants.

Violation of the rank-preserving assumption is possible, although this cannot be tested empirically. Selection bias is a distinct possibility. We know that UK Biobank is subject to extensive selection bias. The response rate for UK Biobank participants was around 5%^11^. Moreover, there is potential for differential selection bias in different age groups and by sex^12^. The UK Biobank recruitment age window was from 40 to 69 years, spanning the typical age of retirement onset. Social and economic factors affecting study participation may be different before and after retirement. Further, at the time of recruitment, retirement ages in the UK were different for men and women, which would further affect selection bias patterns. It is plausible not only that there is selection bias, but that selection bias is differential across strata. NLMR investigations performed in the HUNT dataset, a cohort study based in a particular geographical region of Norway that achieved a response rate of around 70%, showed much weaker evidence of genetic associations with age and sex^10^.

Several of the criticisms of Hamilton *et al* are specific to particular applications of NLMR, rather than criticisms of the method as a whole. They criticize that the results from randomized trials of LDL-cholesterol on coronary heart disease risk differ from NLMR analyses^13^. However, results from the two approaches broadly agree. NLMR suggests that the effect of lowering LDL-cholesterol is to reduce coronary heart disease risk across its distribution – which is in line with the trial evidence. While there are discrepancies in the shape of the curve (trials suggest the curve is slightly convex, NLMR suggests slightly concave), this result is obtained by comparing trial estimates, and so is subject to potential confounding between trials. Additionally, there are differences in demographic characteristics between strata in the NLMR analysis that may lead to different estimates. Notably, another implementation of NLMR for the same causal relationship but with a different choice of genetic instrument suggested a convex curve ^14^. The null findings from the revised NLMR analysis of vitamin D are also in line with trial results^15^. Hamilton *et al* also highlighted discrepant results in the first and second strata of an analysis investigating the potential effect of triglycerides on cancer mortality^13^. However, the overall curve was null, which is in line with trial results. We acknowledge that the doubly-ranked method can give stratum-specific estimates that vary substantially when there are slight changes in the study population; this can be resolved by perturbing the dataset slightly (say, by removing 10 individuals at random), re-running analyses, and averaging across perturbations using Rubin’s rules, as we have done in more recent investigations^10^. The highlighted stratum-specific estimates are anomalous, but given that the publication presented over 100 stratum-specific estimates for different exposures and outcomes^13^, the presence of two odd results is not unexpected.

Hamilton *et al* state that “there should be a pause on further publication of non-linear MR findings”, although in the same sentence, they call for more publications on the topic to find “relevant evidence… that the methods are generating sensible findings”. Investigation of the validity of NLMR will require additional research, both empirical and methodological. Hence a pause in publishing, which is contradicted by the authors themselves, is not warranted. However, there are already lessons that should be learned for future NLMR analyses:

1. NLMR analyses using the residual stratification method are unreliable if the genetic effects on the exposure vary in the population, which is the case for many exposures and cannot be tested reliably by the residual stratification method.
2. Researchers should investigate and report genetic associations with potential confounders and negative controls (including age and sex) both in the population as a whole, and in strata constructed by the stratification method.
3. NLMR analyses should adjust for age and sex (and potentially higher-order terms, such as age-squared and interaction terms) to mitigate potential bias.

A further caution, not mentioned by Hamilton *et al*, is bias from weak instruments. Even if genetic variants are moderately strong in the population as a whole, they may be weaker within strata of the population, due to reduced sample size^16^. Hence if a Mendelian randomization analysis in the population as a whole shows a null result, investigators should be cautious if the NLMR analysis suggests a small positive linear effect, which may reflect the accumulation of small biases in the stratum-specific estimates.

There are several unanswered questions relating to NLMR, which we hope will become clearer as time progresses. Are the genetic associations with age and sex within strata present for all exposures? Can adjustment for age and sex mitigate this bias? Simulation studies have shown that adjustment for predictors of participation in a dataset can reduce selection bias^10^. Hence if the associations demonstrated by Hamilton *et al* are restricted to age and sex, they may not bias estimates substantially in practice, as we can adjust for age and sex. Are these associations present in all datasets? And finally, what is the reason for these associations – violation of the rank-preserving assumption, selection bias, or some other phenomenon?

Currently, our advice to users would be to implement NLMR using the doubly-ranked method for several perturbed datasets averaging across results, to check for associations with age, sex, and other key confounders both overall and within strata (and abandon the investigation if substantial associations beyond those with age and sex are found), and to adjust for age, sex, age-squared, age-sex interaction, and age-squared-sex interaction. Adjustment for variables beyond age and sex could itself lead to collider bias, and so it is not encouraged^17^. This advice is likely to be refined as methodological and empirical investigations continue. While there is intrinsic uncertainty in the assumptions of NLMR and its implementation, there is already intrinsic uncertainty in the application of any Mendelian randomization approach. Although the assumptions for NLMR are stronger than for standard Mendelian randomization, all Mendelian randomization analyses should be viewed with healthy scepticism, and interpreted through the lens of the triangulation framework^18^ as contributing to the evidence basis, not as infallible sources of absolute truth.

Science is an incremental discipline in which new discoveries add to and correct the existing body of scientific knowledge. Each new scientific publication is an admission that our previous knowledge was uncertain, incomplete, or even wrong. As such, the scientific literature is full of papers that are incorrect; this is a feature of science, not a weakness or fault. Of course, we want to minimize errors of both interpretation and fact, but many analyses performed honestly using the best available knowledge at the time of writing are subsequently shown to be erroneous. The answer in such cases is not to stop publishing – but to reflect, learn, correct, and improve. In some cases, retraction may be appropriate, but if every published paper that was subsequently proved to be incorrect in some aspect were retracted, there would be few papers left.
